# Managing Barrett’s esophagus with radiofrequency ablation

**DOI:** 10.1093/gastro/got009

**Published:** 2013-03-08

**Authors:** Junichi Akiyama, Andrew Roorda, George Triadafilopoulos

**Affiliations:** ^1^National Center for Global Health and Medicine, 1-21-1 Toyama, Shinjuku, Tokyo, 162-8655, Japan, ^2^El Camino GI Medical Associates, Mountain View, CA 94040, USA and ^3^Division of Gastroenterology, Stanford University Medical Center, Stanford, CA 94305, USA

**Keywords:** Barrett’s esophagus, radiofrequency ablation, esophageal cancer, adenocarcinoma, intestinal metaplasia, dysplasia

## Abstract

Barrett's esophagus (BE) is a well-established pre-malignant lesion for esophageal adenocarcinoma, a condition that carries a dismal five-year overall survival rate of less than 15%. Among several available methods to eliminate BE, radiofrequency ablation (RFA) provides the most efficient modality, since it has been demonstrated to successfully eradicate BE with or without dysplasia with acceptable safety, efficacy and durability profiles. In conjunction with proton pump therapy, this new technology has quickly become the standard care for patients with dysplastic BE. However, several technical questions remain about how to deploy RFA therapy for maximum effectiveness and long-term favorable outcomes for all stages of the disease. These include how to select patient for therapy, what the best protocol for RFA is, when to use other modalities, such as endoscopic mucosal resection, and what should be considered for refractory BE. This review addresses these questions with the perspective of the best available evidence matched with the authors' experience with the technology.

## INTRODUCTION

Barrett’s esophagus (BE) is a metaplastic condition where the squamous esophageal epithelium is replaced by columnar epithelium containing goblet cells. BE occurs as a result of chronic injury and inflammation of esophageal epithelium due to reflux of gastro-duodenal contents in the context of gastro-esophageal reflux disease (GERD) and its estimated prevalence is about 10% among GERD patients [1]. BE is a well-established pre-malignant lesion for esophageal adenocarcinoma [2, 3]. Adenocarcinoma of the distal esophagus and gastro-esophageal junction (GEJ) have increased in incidence over the last several decades at a rate exceeding that of any other cancer and carry a dismal five-year overall survival rate of less than 15% [4, 5].

Pharmacologic therapy with proton pump inhibitor (PPI) is not only highly effective in relieving acid reflux symptoms in BE, healing of co-existent esophagitis and preventing stricture formation, but also it increases the epithelial differentiation and decreases proliferation of Barrett’s mucosa [6]. Moreover, PPI therapy can lead to partial regression of intestinal metaplasia (IM) in BE patients, as evidenced by the development of macroscopic islands of squamous epithelium and an accompanying shortening of columnar epithelium, albeit inconsistently [7, 9]. A recent large prospective cohort study also showed that PPI use was associated with 75% reduction in the risk of neoplastic progression in patients with BE and that this effect increased with prolonged PPI use and good adherence [10]. However, complete elimination of BE with medical therapy alone is rarely, if ever, accomplished.

Several methods, including thermal ablation (laser, multi-polar electrocoagulation (MPEC), argon plasma coagulation (APC), radiofrequency or cryotherapy), non-thermal ablation (photodynamic therapy), or mechanical (endoscopic mucosal resection) have been introduced to eliminate BE [11]. Of these, radiofrequency ablation (RFA) using the HALO system (Covidien, Sunnyvale, CA, USA) provides the most efficient mode of treatment, since it has been demonstrated to successfully eradicate BE with or without dysplasia with an acceptable safety, efficacy and durability profiles [12–16]. In conjunction with PPI therapy, this new technology has quickly become the standard of care for patients with dysplastic BE.

Ablative therapy with RFA should be considered for patients with dysplastic BE for many reasons: Dysplasia is neoplasia, representing a cancer-cell phenotype confined to the esophageal epithelium. Over many years, this phenotype accumulates a series of oncogenic alterations that enable cellular immortality, cellular autonomy, tissue invasion and other neoplastic characteristics. In patients with HGD undergoing surveillance, the risk of cancer progression is estimated to be approximately six per 100 patient-years during the first few years of follow-up in patients with HGD [17]. Further, the current practice of endoscopic biopsy surveillance is permissive, in that it does not change the natural history of the disease. Endoscopic surveillance also carries a significant sampling error and inter-observer discordance between pathologists, that may erroneously downgrade a patient from high-grade dysplasia (HGD) to low-grade dysplasia (LGD), or LGD to no dysplasia [18]. In contrast, ablative treatment ensures that all neoplastic cells are eradicated. Further, cost-utility models show that ablation is the dominant or preferred strategy for managing dysplastic BE and that alternative management strategies for dysplastic Barrett’s are less optimal [19–21]. Esophagectomy is curative in that it removes the entire organ, yet is fraught with a high rate of morbidity and mortality. Endoscopic resection is technically challenging and is available at few centers with expertise. Overall, the net health benefit (benefit *minus* risk) of RFA for dysplastic BE is favorable and should be available to the patient as a primary option.

A critical question is when to treat BE with RFA based on the disease stage. The American Gastroenterological Association’s (AGA) Medical Position Statement (MPS) recommends RFA treatment for patients with HGD [22]. For LGD, the MPS states that RFA is an appropriate treatment option to be discussed with the patient. There is less clarity around whether to treat patients with non-dysplastic disease: hence, many questions remain about how to deploy RFA therapy for maximum effectiveness and long-term favorable outcomes for all stages of the disease [Table 1]. This review addresses some of these questions with the perspective of the best available evidence matched with the authors’ experience with the technology.

**Table 1.** Current issues regarding radiofrequency therapy for Barrett’s esophagus in clinical practice

Patient selection for radiofrequency therapy
- Which disease stages should physician consider treating? - What clinical characteristics place a non-dysplastic Barrett’s patient at increased risk for neoplastic progression? - Are all patients with Barrett’s esophagus eligible for therapy?
Methods and practice
- What is the best protocol for radiofrequency therapy? - Who should be performing radiofrequency therapy?
Use of other modalities
- How to decide whether to use radiofrequency, endoscopic resection or combined modality therapy? - Which advanced endoscopic imaging is useful for endoscopic surveillance?
Post-therapy surveillance
- What should the follow-up care be?
Refractory disease
- Who is likely to be refractory after radiofrequency therapy? - What should be considered for the radiofrequency-refractory patient?

## PATIENT SELECTION FOR RFA

The AGA MPS states that endoscopic eradication therapy (RFA, PDT or EMR) rather than surveillance is recommended for patients with HGD and should be a therapeutic option for patient with LGD [23]. It also states that, although endoscopic eradication therapy is not suggested for the general population of patients with BE in the absence of dysplasia, RFA with or without EMR should be a therapeutic option for select individuals with non-dysplastic Barrett’s esophagus (ND-BE) who are judged to be at increased risk for progression to high-grade dysplasia or cancer. The recent ASGE guideline recommends endoscopic ablation for BE with HGD; RFA should also be considered and discussed with patients with LGD and for select cases of ND-BE, such as those with a family history of esophageal adenocarcinoma [24]. Given the safety and apparent efficacy of RFA, however, some authorities—including the authors—feel that these guidelines are too restrictive and argue that virtually all patients with BE, irrespective of dysplasia, should be treated with RFA [25].

Although endoscopic surveillance is recommended in patients with BE, with intervals of 3–5 years for ND-BE, 6–12 months for LGD, 3 months for HGD (in the absence of ablation therapy), there are no controlled trials that examine the efficacy of such surveillance. Hence, the question of whether the current strategies to detect and diagnose BE are optimal or justified still remains unanswered. In addition, there has also been a problem with the method of biopsies. Targeted plus 4-quadrant biopsies every 1–2 cm along the length of the BE are recommended but several physicians were especially uncomfortable with surveillance for long-segment Barrett’s esophagus because it is time-consuming and associated with sampling error. There is a need for an adequate, cost-effective screening and surveillance tool to have a significant impact on the rates of adenocarcinoma.

A recent multicenter, sham-controlled trial of RFA achieved complete eradication of dysplasia (CE-D) in 90.5% of patients and complete eradication of IM (CE-IM) in 81% of patients with LGD with 2-year follow-up data demonstrating complete eradication of dysplasia and BE in 98% of patients [15]. The annual rate of neoplastic progression in this study was one per 73 patient-years; however no subjects (sham or ablation) progressed from LGD to cancer [26]. Another multicenter study of RFA of ND-BE achieved complete eradication of BE in 98.4% of patients at 2.5 years and 92% at 5 years, with no patients progressing past ND-BE during follow-up [27]. A recent meta-analysis also demonstrated that endoscopic ablation significantly reduces the risk for cancer in patients with ND-BE and LGD [28]. Moreover, a cost-effectiveness model showed that endoscopic ablation therapy is the preferred strategy over surveillance in patients with ND-BE or LGD [19].

There are a number of reasons why physicians should consider endoscopic intervention rather than ‘surveillance-only’ for patients with ND-BE or LGD: (a) the inability to predict what patients will progress to HGD or invasive cancer, (b) the inability to predict the time course of such progression, should it occur, (c) the risk for misdiagnosis (under-staging) due to inadequate mucosal sampling, lack of compliance with endoscopic surveillance guidelines and inter-observer disagreement between pathologists, (d) the patients’ anxiety for harboring a premalignant lesion and its impact on quality of life and (e) the availability of endoscopic modalities for completely removing the diseased tissue in a safe, effective and cost-effective manner. Therefore, for patients with ND-BE or LGD, RFA plus surveillance or surveillance-alone could be offered after a thorough discussion of the risks and benefits. The risk-benefit discussion should be tailored to each patient’s needs and underlying medical co-morbidities, with a higher emphasis on enrolling higher-risk patients with long-segment BE, a family history of esophageal adenocarcinoma and significant anxiety associated with the diagnosis of BE. In the real-life clinical settings, RFA is now commonly being performed in patients with both dysplastic and non-dysplastic diseases [29]. In fact, because ND-BE is the most common form of BE (95%), RFA is being performed more often in non-dysplastic than dysplastic BE patients.

Although it is generally assumed that BE progresses in a stepwise fashion from ND to LGD to HGD to intra-mucosal cancer and then eventually to invasive cancer, this is unusual in practice. Sharma *et al.* reported that EAC incidence in patients with BE was 0.5% per patient per year of follow-up, but also demonstrated that patients may develop invasive cancer despite having ND-BE as their worst histological grade immediately before being diagnosed with cancer [30].

Differentiating LGD from HGD is challenging and expert discordance in pathology interpretation is frequent and unsettling. This is particularly true for the diagnosis of LGD. Using standard endoscopic imaging—and in the absence of a nodule formation or other mucosal abnormality—it is extremely difficult to distinguish LGD from HGD. Such uncertainty with the diagnosis of dysplasia is enough to drive ablation not only of all dysplasia, but also of any metaplasia. Since we cannot reliably identify who will go on to develop cancer—and in what time frame—and since surveillance is imperfect and economically unsound, ablation for ND-BE and LGD seems even more reasonable to consider. In contrast, there are some circumstances where RFA is difficult and is therefore not recommended. RFA should be avoided in patients with severe co-morbidities, such as cardiopulmonary disease or whose anticoagulation therapy cannot be discontinued.

Formation of a columnar epithelium in the esophagus is the first clinically evident change in the metaplasia-dysplasia-carcinoma sequence, analogous to the precursor status of colon adenomas in the development of colorectal cancer. Hence both BE and colon adenomas represent endoscopically detectable mucosal changes that signify malignant potential. Yet, to date, the clinical management strategies of these conditions have been widely divergent. ND-BE and LGD are approached with watchful endoscopic biopsy surveillance with the goal of detecting disease progression to EAC at a treatable stage. Adenomas, on the other hand, are endoscopically removed upon detection, regardless of histological grade. In both scenarios, patients undergo long-term surveillance at regular intervals.

In order to identify who are the most appropriate candidates for RFA therapy in ND-BE patients, it is important to identify those who are at highest risk of disease progression. Although prospective data are lacking, most authorities agree that the following factors may increase the likelihood of disease progression: long-segment BE (>3 cm) [31–34], large hiatal hernia [32, 35], family history (familial Barrett’s or a primary relative with adenocarcinoma of the esophagus or gastric cardia) [36, 37], male gender [31, 38–40], obesity [41], tobacco use [31, 42, 43], Caucasian race [44, 45], persistent Barrett’s ulcer despite PPI therapy, long-duration or nocturnal GERD [34, 46] and *H. pylori* negative status [47]. With regards to biomarkers, detection of DNA content abnormalities by flow cytometry (aneuploidy or increased 4 N fraction) [48–51], mutation or loss of heterozygosity of the p53 and p16 genes [52–58] and methylation-based biomarkers panels [59, 60] have shown promise and may be superior to histology alone for risk stratification, especially in ND-BE and LGD. However, these biomarkers are not widely available and need to be validated further in large prospective multicenter studies before their routine use in the risk stratification of BE patients can be advocated. Development of a comprehensive BE risk progression score, comprised of both clinical and biomarker variables, should be the ultimate goal and can be achieved by multicenter prospective collaborative efforts. Creation of such a score has the potential to improve outcomes and make the management of patients with ND-BE more cost-effective.

## RFA METHODS AND PRACTICE

For treating long-segment BE, it is best to first use the HALO-360 balloon (circumferential) ablation device then, two months later, check for remaining disease and re-treat as necessary with HALO-90 or HALO-60 focal catheter. Some endoscopists consider this protocol too complex as it requires a sizing process before the actual ablation. They find it quicker and more cost-effective to only use the HALO-90 device for ablation. In our experience, for long-segment BE (>2 cm) and in fairly straight esophageal anatomy, the HALO-360 balloon is definitely the preferred RFA device. In contrast, for disease consisting only of non-circumferential tongues of 1–3 cm (for example, a patient who is C0M2, according to the Prague Classification System) or in patients with large hiatal hernias and tortuous esophageal anatomy, the focal devices—HALO-90 or HALO-60—are preferred.

The advised treatment regimen for HALO-360 procedure comprises two ablation passes with cleaning of both the ablation zone and the ablation balloon after the first pass. This regimen requires multiple introductions and removals of the endoscope, sizing catheter and ablation balloons, which is labor-intensive, time consuming and uncomfortable to the patient. A recent randomized study by van Vilsteren *et al.* showed that the efficacy of a simplified regimen—in which the cleaning step between ablations was completely abandoned—was non-inferior to the standard regimen, but that this regimen was twice as fast (5–13 vs 20 min) and required significantly fewer introductions (RFA devices/endoscope) than the standard approach (4 vs 7) [61]. Hence, the use of this simplified HALO-360 regimen without cleaning step may be considered as a quick and easy alternative for patients with an uncomplicated BE without scarring and luminal stenosis.

A large number of RFA clinical trials have shown high efficacy and safety rates to treat BE but such trials have been conducted at predominantly academic tertiary care centers. Lyday *et al.* reported that the safety and efficacy outcomes of RFA performed at community-based practice were comparable to those previously reported in multicenter trials from predominantly tertiary academic centers [62]. The main strengths of the HALO system include the precise and controlled delivery of a predetermined, standardized radiofrequency energy and its simplicity and ease, since it can be performed in a less operator-dependant manner. Hence, RFA may be performed by any clinician who has an in-depth understanding of esophageal pathophysiology, who has the equipment and expertise to perform adequate BE imaging (preferably with high-definition endoscope) and who is willing to make long-term commitment to the BE patient by performing surveillance adhering to the Society guidelines in order to assure durability of eradication. Although accurate staging is critical in making therapeutic decisions in patients with dysplastic BE, physicians performing RFA do not necessarily need to have EMR and EUS capabilities if there is an intention to refer challenging cases to an expert BE center [63].

## USE OF OTHER MODALITIES

The primary goal of endoscopic treatment in BE is to prevent the development of invasive esophageal adenocarcinoma. There are currently two main endoscopic techniques employed for BE eradication: tissue ablation and tissue resection (EMR). The principle behind all ablative techniques is the superficial induction of necrosis of the metaplastic and dysplastic tissue. Cellular damage can be produced through thermal injury (RFA), photochemical injury (photodynamic therapy) or exposure to cold temperatures (cryotherapy). The choice of ablative therapy is somewhat empiric, given the lack of comparative studies looking at long-term outcomes with different techniques. RFA may be best suited for flat mucosa, where the ablation catheter can establish direct contact with the entire mucosa. Treatment in patients with scarring and distorted anatomy, which makes contact challenging, may be approached with cryoablation. PDT use has declined due to prolonged photo-sensitivity and a high rate of stricture formation.

As in most malignancies, EAC survival correlates with cancer staging, so accurate initial clinical staging is crucial. The first clinical assessment to make when EAC is diagnosed is the differentiation of intramucosal cancer (IMC) from submucosal (or deeper) adenocarcinoma. In general, routine biopsies are not adequate to provide a reliable pathological assessment of the depth of tumor invasion in order to make a safe clinical decision about utilization of local treatment alone. To address this limitation, both endoscopic ultrasound (EUS) and EMR have been applied. EUS is currently regarded as the best local staging tool available for esophageal cancer. However, EUS is incapable of reliably differentiating between T1a and T1b tumors and, therefore, it cannot reliably stratify patients suitable for local rather than surgical treatment; but it is used to rule out lymph node metastases, as a suspicious lymph node can be detected and biopsied for cytological evaluation. In contrast to EUS, EMR provides large tissue specimens that can be used to assess the depth of any neoplastic involvement and the adequacy of the resection.

EMR is also used to remove focal, nodular areas of dysplasia and neoplasia within the BE segment. Thus, EMR has potential value as a diagnostic/staging procedure, as well as a therapeutic procedure for removing Barrett’s epithelium with dysplasia. Although EMR removes any visibly suspicious areas and has the advantage of allowing histological evaluation of the specimen with subsequent risk stratification of the specimen for the presence of lymph node metastases, this leaves the patient at potential risk of metachronous lesions during follow-up. To prevent this, EMR has been combined with RFA to eradicate residual metaplasia. However, RFA after EMR could theoretically result in an increase in the incidence of procedural complications; scarring of the esophagus and change in compliance of the esophageal wall after EMR might make the performance more challenging and potentially increase the risk of perforations, tears and stricture formation. Recent studies have reported that, in patients with EMR before RFA for nodular BE with HGD or IMC, no differences in efficacy and safety outcomes were observed, compared with RFA alone, for non-nodular BE with HGD or IMC [64, 65]. EMR followed by RFA is a safe and effective treatment strategy for patients with nodular BE and advanced neoplasia.

A recent systematic review, that included 1874 patients with HGD and mucosal cancer undergoing esophagectomy, showed overall risk of lymph node metastasis of 1.39% (95% CI: 0.86–1.92) [66]. In contrast, in patients with submucosal invasion, the rate of lymph node metastasis is substantially higher (20–30%) [67]. The risk factors predictive of tumor recurrence or lymph node spread include moderate-to-poor tumor differentiation, evidence of vascular lymphatic or neural invasion and the presence of multifocal HGD [68]. Esophagectomy should be employed in patients with submucosal invasion or at risk with lymph node involvement, determined by staging EMR or EUS, or when patients cannot comply with post-treatment endoscopic surveillance after endoscopic treatment for HDG or intra-mucosal cancer. A decision on endoscopic therapy vs resective surgery should be made following thorough discussion of the advantages and disadvantages of each approach. Institutional endoscopic and surgical expertise, as well as patient preferences and risk tolerance, will likely influence choice of therapy.

Given the patchy and focal distribution of dysplasia within the Barrett’s segment, advanced endoscopic imaging technologies—such as high definition (HD) endoscopy, narrow band imaging (NBI), autofluorescence imaging (AFI) and confocal laser endomicroscopy (CLE)—have been developed to enhance detection of dysplasia and early cancer in BE. Some of these technologies (HD-WLE, AFI) are designed for *detection* of abnormalities, while other imaging modalities are better suited for tissue *characterization* (NBI, chromoendoscopy) and histological confirmation (CLE). HD-WLE has a higher sensitivity for the detection of Barrett’s-related neoplasia compared to standard endoscopy and is now the standard of care.

NBI allows for better detection and characterization of early neoplasia and IM by highlighting mucosal surface structures and microvasculature. It has been demonstrated that NBI can detect significantly more patients with dysplasia and higher grades of dysplasia with fewer biopsy samples compared with standard resolution WLE [69]. When compared to HD-WLE, the results have been less encouraging. Kara *et al.* found that targeted biopsies with HD-WLE alone had a sensitivity of 79% for the detection of HGD and the addition of NBI did not result in the additional detection of HGD/EAC [70]. Sharma *et al.* also reported that there was no difference between HD-WLE with four-quadrant biopsies and NBI-directed biopsies in the overall detection of dysplasia (55 vs 77%; *P* = 0.15), but NBI required fewer biopsies per procedure to establish the diagnosis (3.6 vs 7.6; *P* < 0.0001) [71]. On the other hand, NBI with magnification has been used to characterize and distinguish lesions detected by HD-WLE. A meta-analysis showed that NBI with magnification has high diagnostic precision in detecting HGD with a sensitivity and specificity of 96% (95%CI; 93–99) and 94% (95%CI; 84–100), respectively [72]. However, the results of NBI with magnification in characterizing SIM were inferior with a sensitivity of 95% (95%CI; 87–100) and a specificity of 65% (95% CI; 52–78). A uniform classification system is needed for the evaluation of mucosal and vascular patterns in BE with NBI with magnification and this classification needs to be validated, incorporating intra-observer and inter-observer agreement as well. Endoscopic tri-modal imaging (ETMI) (not available in the USA) that includes HD-WLE, AFI and NBI with magnification, has shown no added benefits in the detection of dysplasia [73, 74].

CLE is one of the newest endoscopic technologies, which provides an *in vivo* microscopic assessment of BE mucosa. Currently there are two confocal platforms available for clinical use: endoscope-based CLE (eCLE) and probe-based CLE (pCLE) [75]. Recently, Sharma *et al.* published results of a multicenter, randomized, controlled trial to determine the accuracy of pCLE in real time. Patients were examined by HD-WLE, NBI and pCLE. The sensitivity and specificity for HD-WLE were 34.2% and 92.7%, respectively, compared with 68.3% and 87.8%, respectively, for HD-WLE with pCLE (*P* = 0.002 and *P* < 0.001, respectively). Development and validation of criteria for this technology in the detection of HGD/EAC was described with a high overall accuracy and short learning curve [76]. Unfortunately, data are lacking on standardized diagnostic criteria and differentiating LGD from ND-BE or HGD using these advanced imaging tools. Hence, the AGA MPS does not recommend use of these advanced imaging techniques for routine surveillance endoscopy.

## POST-RFA SURVEILLANCE

Once BE has been removed by RFA, patients need follow-up surveillance to monitor disease recurrence. Histological assessment of the post-RFA esophagus is performed by surveillance endoscopy with systematic biopsies of the neo-squamous epithelium. The AGA MPS does not specify a protocol for how often patients should be brought back for further upper endoscopy examination, but most authorities agree that post-RFA surveillance (endoscopy with biopsies) should be performed at pre-RFA intervals based on the prior presence of dysplasia and its grade. Hence, HGD should be surveyed every three months, then every six months and yearly thereafter. Low-grade dysplasia should be surveyed every six months, then one year later and every one to five years thereafter. ND-BE should be surveyed every three to five years. Surveillance for any stage of disease should not be continued indefinitely but only until a patient’s advanced age or co-morbidities suggested no further utility. Biopsies should be done according to the Seattle protocol; cardiac biopsies should also be taken but labeled separately. It is also commonly accepted that long-segment BE and presence of nodules should shorten surveillance intervals.

There have been several reports regarding the long-term outcomes and durability of the neo-squamous epithelium induced after RFA therapy [26, 77–82]. These studies showed that ≥80% were able to maintain CE-D and CE-IM without additional therapy. The recurrence rate of IM and dysplasia is low and, among those with recurrent IM, most are histologically worse than the pretreatment grade and the area of recurrence is generally small. Recurrent IM is usually recognized in three distinct patterns: endoscopically invisible IM underneath the neosquamous epithelium (buried glands), visible recurrence in the tubular esophagus (recurrent tongues) and IM of GEJ (with a squamous-lined tubular esophagus) [83].

Buried glands after RFA are uncommon and have been found in less than 10% of patients enrolled in clinical trials evaluating RFA. A systematic review of 18 reports showed that buried metaplasia was noted in nine (0.9%) of the 1004 patients after RFA [84]. The buried glands are not visible by conventional endoscopy, even when supplemented by chromogen or narrow-band imaging. Although rigorous biopsies were performed over neosquamous epithelium after RFA treatment in study protocols, these buried glands are likely underappreciated with the current surveillance protocols because areas of neosquamous epithelium do not routinely undergo biopsy in clinical practice. Furthermore, the sampling area and depth of biopsy are limited, even with large-capacity forceps.

Optical coherence tomography (OCT) is an emerging biomedical imaging technology that detects light back-scattered from tissues to construct cross-sectional and three-dimensional (3D) images of tissue microstructures with micron-scale resolution. OCT imaging depth is 1 to 2 mm in the human esophagus, enabling evaluation of tissue morphology underneath the squamous epithelium. Because the location of buried glands is unpredictable, this imaging technology, which uniquely enables depth-resolved imaging of a broad area with near-microscopic resolution, holds great potential for identifying and characterizing buried glands before and after ablative therapies. Zhou *et al.* reported that 3D-OCT provided a 30 to 60 times larger field of view, compared with jumbo and standard forceps biopsy, and sufficient imaging depth to the lamina propria/muscularis mucosa to facilitate the detection of buried glands before and after RFA. It also detected a high prevalence of buried glands in 72% patients before RFA and in 63% patients after RF, although the number of buried glands per patient was significantly lower after RFA [85].

Only a few studies have evaluated the biological properties of buried metaplasia. Buried metaplasia following PDT appears to have a lower crypt proliferation rate and neoplastic potential compared with pretreatment BE [86]. A recent report raised concerns on the development of sub-squamous neoplasia in three patients who were treated with RFA for BE (two developed adenocarcinoma and one developed HGD) [87]. Further investigation is needed to understand the longitudinal progression and clinical implications of buried glands.

The predictors for recurrence of BE have also not been well understood. In one study by Vaccaro *et al.*, a longer baseline BE length was shown to be associated with IM recurrence [81]. The reasons for such an association are unclear but longer length may be a marker of more severe reflux, or may be a surrogate measure of the likelihood of harboring a more genetically advanced dysplastic clone. Further studies are needed to identify predictors of recurrence, which would be valuable to risk-stratify patients after ablation to better target surveillance efforts.

## REFRACTORY DISEASE

There are several factors associated with incomplete response to RFA. These include longer columnar segment, large hiatal hernia and ongoing uncontrolled reflux. The length of initial columnar segment may influence the ability to perform a successful ablation and the number of ablations needed to achieve success [15, 63, 88–90]. Although the increased surface area of the columnar mucosa may be responsible for this observation, another possible explanation may be that patients with longer columnar segments may have more severe reflux. Hiatal hernia size is also known to affect the ability to successfully ablate BE [63, 88, 91]. The widening of the distal esophagus into a hiatal hernia may make it difficult to bring the electrode of the HALO circumferential/focal devices into good contact with the mucosa at the GEJ, resulting in insufficient ablation of BE at this level.

Uncontrolled reflux, despite twice daily PPI therapy, is also reported to increase the incidence of persistent IM after ablation in patients with BE [88, 91, 92]. Persistent esophageal acid reflux without symptoms is frequently observed among BE patients treated with PPI, or even fundoplication [91, 93–95]. In RFA clinical trials, patients typically receive double-dose PPI without evaluating their actual amount of acid reflux by pH/impedance monitoring. However, a significant number of BE patients will not demonstrate normalization of gastro-esophageal reflux, even when taking high-dose PPI therapy [95]. The potential explanation for this observation could be incompetent lower esophageal sphincter and ineffective esophageal motility [96], reduced chemo- and mechanoreceptor sensitivity to acid or balloon distension [97, 98] and reduced symptom perception in BE patients [99].

The number of RFA sessions required to eradicate BE and associated dysplasia is usually 2–3, but more sessions may be needed for longer BE segments [62]. There is no consensus on the number of treatments needed to define a non-responder or a refractory patient after several RFA sessions.

These patients may be considered to perform esophageal function testing, such as impedance, pH testing and/or high-resolution manometry to assess for uncontrolled reflux despite therapy. If the reflux testing shows ongoing reflux despite PPI therapy, laparoscopic fundoplication should be considered, since it may correct both reflux and underlying hiatal hernia, which are the major underlying causes responsible for incomplete response to RFA [89].

There have been several reports addressing no response after RFA, which is endoscopically characterized by replacement of the ablated area with a scar but without squamous regeneration. After 2 more months of follow-up, these patients heal with recurrent columnar lining in the ablated segment. This phenomenon is not well described in the literature but such patients may account for 9–15% of cases [63, 77, 79]. In one study, even after the fundoplication was carried out and resulted in normalization of intra-esophageal pH, two out of three patients still could not be successfully ablated [63]. Further study will be needed to assess the underlying mechanisms behind refractory disease other than ongoing acid reflux.

## CONCLUSIONS

Significant evidence has been accumulated recently that supports the use of RFA in dysplastic and non-dysplastic BE but several technical questions remain. In general, however, the HALO systems have provided a safe and practical approach to BE management at the level of community practice and are expected to have a favorable impact on esophageal adenocarcinoma rates.

**Conflict of interest:** G.T. has received consulting honoraria from BARRx Medical, Inc.
